# Association between CD8+ Tumor Infiltrating Lymphocytes and the Clinical Outcome of Patients with Operable Breast Cancer Treated with Adjuvant Dose-Dense Chemotherapy—A 10 Year Follow-Up Report of a Hellenic Cooperative Oncology Group Observational Study

**DOI:** 10.3390/cancers14225635

**Published:** 2022-11-16

**Authors:** Nikolaos Spathas, Anna C. Goussia, Georgia-Angeliki Koliou, Helen Gogas, Flora Zagouri, Anna Batistatou, Antonia V. Charchanti, Alexandra Papoudou-Bai, Mattheos Bobos, Sofia Chrisafi, Kyriakos Chatzopoulos, Ioannis Kostopoulos, Triantafyllia Koletsa, Petroula Arapantoni, Dimitrios Pectasides, Eleni Galani, Angelos Koutras, George Zarkavelis, Emmanouil Saloustros, Dimitrios Bafaloukos, Charisios Karanikiotis, Iliada Bompolaki, Gerasimos Aravantinos, Amanda Psyrri, Evangelia Razis, Anna Koumarianou, Eleni Res, Helena Linardou, George Fountzilas

**Affiliations:** 1 Fourth Oncology Department, Metropolitan Hospital, 18547 Athens, Greece; 2 Department of Pathology, Ioannina University Hospital, 45500 Ioannina, Greece; 3 Section of Biostatistics, Hellenic Cooperative Oncology Group, Data Office, 11526 Athens, Greece; 4 First Department of Medicine, Laiko General Hospital, National and Kapodistrian University of Athens School of Medicine, 11527 Athens, Greece; 5 Department of Clinical Therapeutics, Alexandra Hospital, National and Kapodistrian University of Athens School of Medicine, 11528 Athens, Greece; 6 Laboratory of Molecular Oncology, Hellenic Foundation for Cancer Research/Aristotle University of Thessaloniki, 54006 Thessaloniki, Greece; 7 Department of Pathology, School of Health Sciences, Faculty of Medicine, Aristotle University of Thessaloniki, 54124 Thessaloniki, Greece; 8 Department of Pathology, Henry Dunant Hospital, 11526 Athens, Greece; 9 Oncology Section, Second Department of Internal Medicine, Hippokration Hospital, 11527 Athens, Greece; 10 Second Department of Medical Oncology, Metropolitan Hospital, 18547 Piraeus, Greece; 11 Division of Oncology, Department of Medicine, University Hospital, University of Patras Medical School, 26504 Patras, Greece; 12 Department of Medical Oncology, Medical School, University of Ioannina, 45500 Ioannina, Greece; 13 Department of Oncology, University Hospital of Larissa, 41334 Larissa, Greece; 14 First Department of Medical Oncology, Metropolitan Hospital, 18547 Piraeus, Greece; 15 Department of Medical Oncology, 424 Army General Hospital, 56429 Thessaloniki, Greece; 16 Oncology Department, General Hospital of Chania, 73300 Crete, Greece; 17 Second Department of Medical Oncology, Agii Anargiri Cancer Hospital, 14564 Athens, Greece; 18 Section of Medical Oncology, Department of Internal Medicine, Attikon University Hospital, Faculty of Medicine, National and Kapodistrian University of Athens School of Medicine, 12462 Athens, Greece; 19 Third Department of Medical Oncology, Hygeia Hospital, 15123 Athens, Greece; 20 Hematology-Oncology Unit, Fourth Department of Internal Medicine, Attikon University Hospital, National and Kapodistrian University of Athens School of Medicine, 12462 Athens, Greece; 21 Third Department of Medical Oncology, Agii Anargiri Cancer Hospital, 14564 Athens, Greece; 22 Aristotle University of Thessaloniki, 54124 Thessaloniki, Greece; 23 Department of Medical Oncology, German Oncology Center, 4108 Limassol, Cyprus

**Keywords:** biomarkers, tumor-infiltrating lymphocytes, breast cancer, adjuvant chemotherapy

## Abstract

**Simple Summary:**

Tumor-infiltrating lymphocytes (TILs) have been reported to contribute to breast cancer (BC) prognosis. The aim of our study was to investigate the prognostic impact of CD8+ TILs and their subtypes in patients with early breast cancer treated with sequential, dose-dense adjuvant chemotherapy. Tumors of 627 patients were examined for total (t), stromal (s), and intratumoral (i) CD8 lymphocyte density (counts/mm^2^). Our results showed that high expression of sCD8, iCD8, and tCD8 correlated with higher Ki67, TILs density, ER/PgR negativity, and higher histological grade. We confirmed that patients with high iCD8+ and tCD8+ TILs had longer DFS and OS, as compared to those with low counts/mm^2^. Survival benefit was retained when adjusting for classical clinical and pathological characteristics, but was not correlated to specific BC subtype. More data are needed to empower the prognostic role of TILs and establish their use in clinical practice.

**Abstract:**

Tumor-infiltrating lymphocytes (TILs) contribute to breast cancer (BC) prognosis. We investigated the prognostic impact of CD8+ TILs in patients with early breast cancer treated with adjuvant chemotherapy in a large observational clinical trial. Along with a 10 year follow-up, considering the efficacy and safety, we report the results of the translational part of our study. We examined the patients’ tumors for total (t), stromal (s), and intratumoral (i) CD8 lymphocyte density (counts/mm^2^) on tissue-microarray cores. The impact of CD8+ TILs counts on DFS and OS, and its correlation with breast cancer subtypes and standard clinicopathological parameters, were investigated, along with efficacy and safety data. Among the 928 eligible patients, 627 had available CD8+ data. Of which, 24.9% had a high expression of sCD8, iCD8, and total CD8, which were correlated with higher Ki67, TILs density, ER/PgR negativity, and higher histological grade. The 5year DFS and OS rates were 86.1% and 91.4%, respectively. Patients with high iCD8 and tCD8 had longer DFS and OS compared to those with low counts/mm^2^ (DFS: HR = 0.58, *p* = 0.011 and HR = 0.65, *p* = 0.034 and OS: HR = 0.63, *p* = 0.043 and HR = 0.58, *p* = 0.020, respectively). Upon adjustment for clinicopathological parameters, iCD8 and tCD8 retained their favorable prognostic significance for DFS and OS, whereas high sCD8 was only prognostic for DFS. Menopausal status, tumor size, and nodal status retained their prognostic significance in all examined multivariate models. CD8+ TILs, and especially their intratumoral subset, represent a potential favorable prognostic factor in early BC.

## 1. Introduction

Female breast cancer (BC) is the most commonly diagnosed cancer globally and represents the leading cause of cancer death among women [1]. Surgery, with the addition of adjuvant/neoadjuvant systemic therapy, is the gold standard for the treatment of early stage breast cancer and leads to an increase in the patients’ survival rate [2]. Traditionally, tumor grade, tumor size, lymph node status, estrogen (ER), progesterone (PgR), and human epidermal growth factor (HER2/neu or c-erbB2) receptor status have represented the main prognostic markers for BC [3]. Despite important improvements in early BC diagnosis and treatment, new biomarkers are needed, to further improve outcomes and reduce recurrence rates.

The tumor immune microenvironment has been reported to play a vital role in cancer spread, progression, and response to treatment [4], and several studies have investigated its role in BC [5,6]. Tumor infiltrating lymphocytes (TILs), an integral element of the tumor immune microenvironment, have been studied in several malignancies, concerning their prognostic/predictive value [7,8]. TILs and their subtypes have been widely investigated in the field of BC and have demonstrated a proven prognostic [9,10] and predictive role [11,12]. A major sub-population of TILs, cytotoxic CD8 + T cells, are reported to be a favorable prognostic factor [13,14], although their impact on prognosis seems to differ between the various clinicopathological subtypes of BC [15,16]. The location of TILs (stromal TILs (TILs) or intratumoral TILs (iTILs)) has also been reported to be an independent factor associated with BC outcomes. Similarly, the location of CD8+ TILs seems to play a prognostic role in certain types of BC [17].

Beyond prognosis, there has been a large research effort to establish TILs as predictive markers and establish their role in daily clinical practice. Losurdo et al. showed that low TILs negatively impact prediction of outcome in TNBC treated with immunotherapy. This information may suggest a need for induction chemotherapy before PD1/PD-L1 [18] inhibitors in this specific subpopulation. Another study suggested that TILs may be useful as a predictive marker of the therapeutic effect of eribulin chemotherapy in TNBC [19]. More recently, TILs have found their place in cancer treatment. Emerging data showed that adoptive cell therapy with TILs can be an effective treatment for metastatic melanoma [20]. Similarly, a recently published study demonstrated that cell therapy with autologous TILs may constitute a new treatment strategy in metastatic lung cancer [21].

Despite these meaningful advantages in the field, the prognostic or predictive value of TILs in BC remains debatable, due to the significant amount of heterogeneity in the experimental design. In particular, the variability of the methods and criteria used to quantify TILs and the interobserver differences between pathologists when evaluating iTILs constitute obstacles to the integration of TILs into clinical practice. In addition, the heterogeneity of BC makes the use of TILs much more complex [22,23,24].

The aim of the present study was to evaluate the prognostic role of CD8+ TILs within a Hellenic Cooperative Oncology Group study, along with a final report on the efficacy and safety data after a 10 year follow-up period.

## 2. Results

### 2.1. Patient and Tumor Characteristics

**Figure 1:**
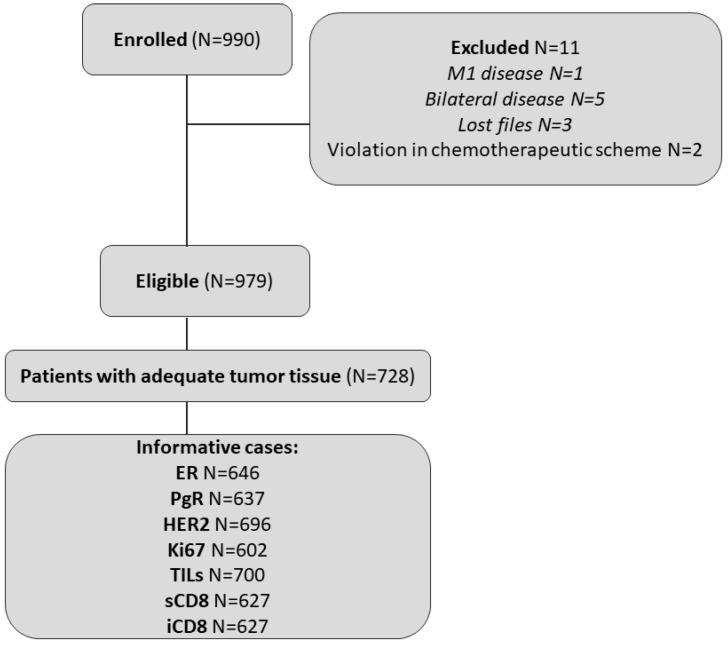
REMARK diagram.

A total of 990 women were enrolled in the study between November 2007 and December 2010. Eleven patients were deemed ineligible and were excluded from the analysis (Figure 1), leading to a total of 979 eligible patients. Among these, 728 (74.4%) had consented to the use of their biological material for future research.

Selected patient and tumor characteristics are presented in Table 1. The median age at diagnosis was 54 years (range 28–83). About half of the women were postmenopausal (57.6%), had undergone modified radical mastectomy (55.7%), and carried tumors of 2.1–5 cm (51.7%). A central assessment of ER/PgR and HER2 status was available for 88.3% (n = 643) and 95.6% (n = 696) of patients with evaluable FFPEs, respectively. The discordance rate between the local and central assessment was 11.4% for ER/PgR and 8.9% for HER2. Luminal A tumors were classified in 24.5%, luminal B in 44.1%, luminal HER2 in 12.9%, HER2-enriched in 7.1%, and triple-negative (TNBC) in 11.4% of informative cases (n = 588), based on the central assessment. Additionally, CD8 data were available for 86.1% of patients with evaluable FFPEs (n = 627 patients). No significant differences were noted in basic patient and tumor characteristics between the total cohort of eligible patients (n = 979) and patients with evaluable FFPEs for translational research (n = 728).

### 2.2. Drug Exposure

The relative dose intensities of all drugs were assessed in the 964 patients (98.5%) with available data who received the study treatment. Of note, five patients received docetaxel instead of paclitaxel, while one additional patient was treated with adriamycin instead of epirubicin. Lastly, another patient received epirubicin followed by the administration of doxorubicin.

The median relative dose intensity (RDI) administered for epirubicin and paclitaxel was 0.997 and 1.00, respectively. This was similar to previously published results by our group [25,26,27]. The median RDI for cyclophosphamide, fluorouracil, and methotrexate was 0.714, 0.714, and 0.785, respectively. Eight patients discontinued treatment with paclitaxel due to allergic reactions/intolerance and continued treatment with docetaxel. Treatment completion was achieved in 902 of 964 patients (93.6%). The main reasons for the discontinuation of study treatment included non-fatal toxicity (n = 35; 56.5%), followed by refusal to continue (n = 11; 17.7%), doctor’s decision (n = 7; 11.3%), moving to another hospital (n = 5; 8.1%), and other reasons (n = 4; 6.5%). Trastuzumab was administered in 214 of 225 patients (95.1%) with HER2-positive disease, according to the local assessment. Additionally, one patient with HER2-negative disease received trastuzumab, due to ambiguous CISH results.

### 2.3. Immunohistochemical Findings

**Figure 2:**
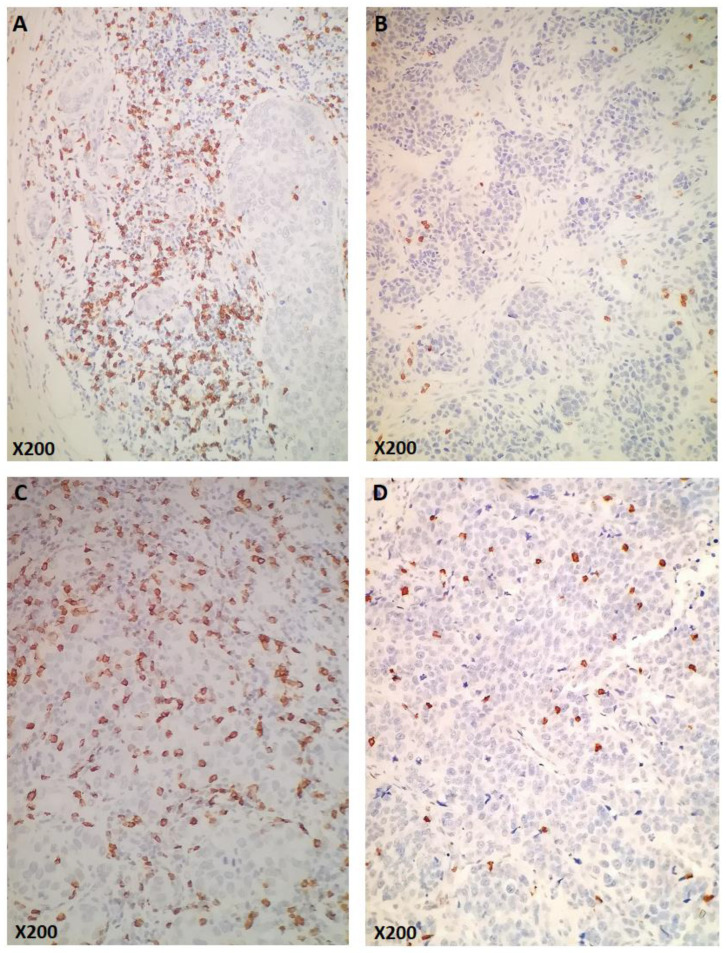
Examples of CD8 immunohistochemical expression (original magnification as indicated). (**A**,**B**): Cases representing high (**A**) and low (**B**) numbers of stromal CD8 (sCD8+). (**C**,**D**): Cases representing high (**C**) and low (**D**) numbers of intratumoral (intraepithelial) CD8 (iCD8+).

The median number of sCD8 and iCD8 counts/mm^2^ was 113.7 and 3.7, respectively (Supplemental Figure S1). Of 627 tumors with available CD8 data, 156 (24.9%) had high expression of sCD8, iCD8, and tCD8 (Figure 2A–D). Patients carrying tumors with high counts/mm^2^ of all lymphocytic subsets (including stromal, intratumoral, and total CD8) had higher Ki67 levels (all *p*-values < 0.001) and higher TILs density (all *p*-values < 0.001). Additionally, tumors with high counts/mm^2^ of sCD8, iCD8, and tCD8 were more frequently ER/PgR-negative (all *p*-values < 0.001) and of higher histological grade (all *p*-values < 0.001) (Supplemental Table S2). TILs density was strongly, positively correlated with sCD8 (rho = 0.46, *p* < 0.001), iCD8 (rho = 0.50, *p* < 0.001), and tCD8 (rho = 0.50, *p* < 0.001). Additionally, a significant positive correlation was observed between TILs density and Ki67, which, however, was not considered strong (rho = 0.32, *p* < 0.001).

### 2.4. Efficacy

At a median follow-up of 132.5 months (range 6.8–158.8), 253 DFS events (25.8%) had been recorded and 201 deaths (20.5%) had occurred. Information on the cause of death was available for 140 of 148 patients (94.6%) who died upon disease progression following adjuvant E-T-CMF chemotherapy, with 96.4% of them (n = 135) dying of their disease. No deaths were recorded during adjuvant chemotherapy. The median DFS and OS had not yet been reached at the data cut-off date for the analysis (15 June 2021). The 5-year DFS and OS rates were 86.1% (95% CI 84–88%) and 91.4% (95% CI 90–93%), respectively. Four patients (0.4%) developed myelodysplastic syndrome and 33 patients (3.4%) developed other cancers, including colorectal cancer (n = 1), endometrial cancer (n = 3), gastric cancer (n = 2), lung cancer (n = 5), breast cancer (n = 10), ovarian cancer (n = 3), pancreatic cancer (n = 3), melanoma of the skin (n = 3), sarcoma (n = 1), non-Hodgkin lymphoma (n = 1), and multiple myeloma (n = 1). In addition, two patients (0.2%) developed in situ breast carcinoma, seven months and 10 years post-completion of adjuvant chemotherapy, respectively.

Data regarding the site of progression were available for 163 of 185 patients (88.1%) who experienced disease progression. Distant relapses were noted in 151 patients (15.4% of the total cohort; 92.6% of progressors), while 19 patients (1.9% of the total cohort; 11.7% of progressors) presented locoregional relapses. Of note, six patients experienced both locoregional and distant relapses (Supplemental Table S3).

As expected, increasing age, postmenopausal status, radical mastectomy, increased tumor size and higher nodal status were associated with worse patient outcomes in terms of both DFS and OS. In addition, patients treated with adjuvant radiotherapy were at a significantly higher risk of death compared to those who did not receive radiation (Supplemental Table S4).

**Figure 3:**
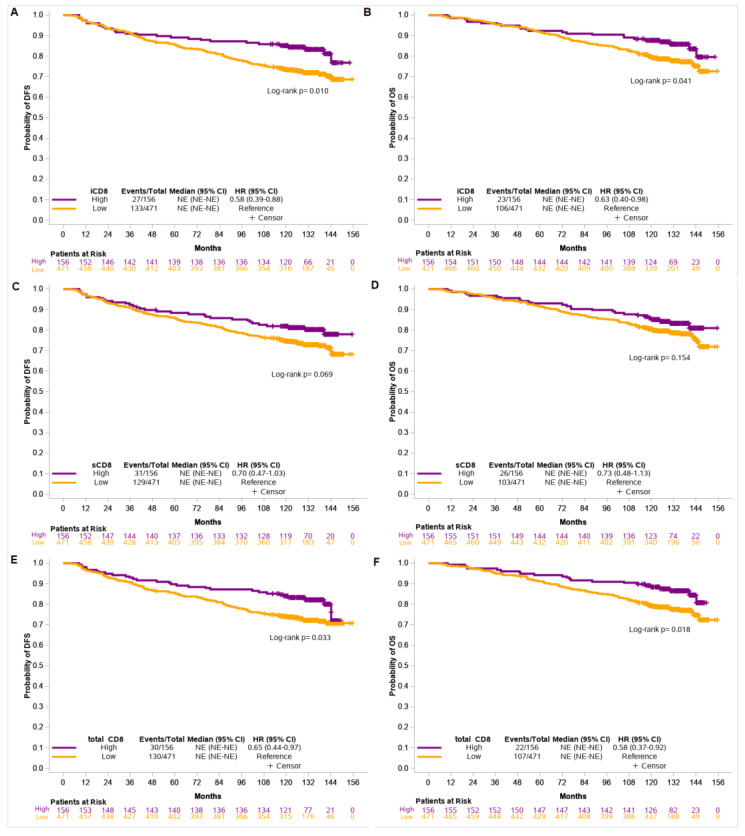
Disease-free survival (DFS) and overall survival (OS) based on iCD8 (**A**,**B**), sCD8 (**C**,**D**), and total CD8 (**E**,**F**) counts/mm^2^ in the entire cohort of patients.

Patients with high counts/mm^2^ of iCD8 and tCD8 had longer DFS and OS compared to those with low counts/mm^2^ (DFS: HR = 0.58, 95% CI 0.39–0.88, *p* = 0.011 and HR = 0.65, 95% CI 0.44–0.97, *p* = 0.034 and OS: HR = 0.63, 95% CI 0.40–0.98, *p* = 0.043 and HR = 0.58, 95% CI 0.37–0.92, *p* = 0.020, respectively) (Figure 3).

TIL density was not associated with patients’ outcome, while only a trend towards improved DFS was observed for tumors with high counts/mm^2^ of sCD8 (HR = 0.70, 95% CI 0.47–1.03, *p* = 0.070) (Supplemental Table S5).

Upon adjustment for selected clinicopathological parameters (as described in the statistical analysis section), both iCD8 and tCD8 retained their favorable prognostic significance for DFS and OS, whereas high counts/mm^2^ of sCD8 were significantly associated with prolonged DFS (Table 2). Of note, menopausal status, tumor size, and nodal status retained their prognostic significance for DFS and OS in all examined multivariate models.

We further attempted to assess the prognostic significance of the markers of interest in the subgroups of patients defined by molecular subtype. However, due to the absence of, or the extremely small number of, events of interest in the category of patients with high counts/mm^2^ of sCD8, iCD8, and tCD8, the evaluation of the impact of CD8 expression on DFS and OS was only feasible among patients with luminal B tumors, but neither CD8 nor TIL density showed prognostic significance in this subpopulation (Supplemental Table S5).

### 2.5. Safety Profile

The safety profile was assessed in 944 patients with available data (96.4%). Among these, 226 patients reported a total of 325 grade 3–4 events (Table 3). No fatal adverse events were recorded. Neutropenia was the most commonly reported adverse event throughout the study treatment (65 grade 3–4 adverse events), followed by neuropathy (45 severe adverse events).

## 3. Discussion

In the present study, we investigated the impact of CD8+ TIL density on the survival of patients with early BC treated with adjuvant chemotherapy and its correlation with classical clinicopathological characteristics. Simultaneously, we documented the first report of a HeCOG clinical study, regarding the efficacy and safety results after a 10-year follow-up period.

Patients enrolled in this study were treated with an E-T-CMF regimen followed as indicated by one year of trastuzumab treatment. This regimen was used in several studies by our group [25,26,27]; variations existed in the use of taxanes, as at the time of the design of this trial, the optimal dosing schemes and preferable type of taxanes were under investigation [28,29]. Although as dose-dense chemotherapy had already been shown to be superior to conventionally scheduled regimens [30,31], we decided to conduct chemotherapy on a biweekly schedule. Trastuzumab was given for one year in patients with HER2- positive tumors, as it had been recently correlated in several trials with survival benefit in this subgroup [32,33,34].

After a 10 year follow-up, it was found that a DFS event occurred in 25.8% of patients and that 20.5% of patients died of various reasons. The 5 year DFS and OS rates were 86.1% and 94.1%, respectively. These results were consistent with the efficacy data of other reported adjuvant trials by our research group [25,26,27] and with pivotal international randomized trials [30,35,36,37]. Similarly, the rates of secondary malignancies, sites of progression, and disease characteristics associated with worse outcomes were, to a large extent, as expected.

As mentioned above, in this trial, we investigated TIL density and the presence of CD8+ TILs in a series of BC patients, their correlation with specific clinicopathological parameters, and their correlation with patient outcomes. High CD8+ TIL counts, regardless of subtype, were associated with tumors demonstrating higher TIL density, higher ki67, and higher tumor grade. Κi67 index is not T cell specific but an indicator of proliferation activity of tumor cells. Therefore, a high Ki67 index provides an estimation of the growth fraction of tumor cells and reflects the ability of neoplastic cells to highly proliferate. In breast cancer, Ki67 immunohistochemical expression has been extensively investigated and is applied to assess the proliferative activity of cancer cells, taking account of breast cancer molecular subtyping. As it seems that a favorable prognostic factor correlates with poor prognostic parameters, our findings may, in fact, reflect that, in tumors with similar ki67 or the same histological grade, higher TILs can play a favorable prognostic role, if present. In the statistical analysis of this study, ki67 was not examined regarding survival and after multivariate regression, and histological grade was related with improved OS in high CD8+ tumors. The methodology we followed, in combination with the small size of CD8 positive tumors in our study, suggest that the prognostic significance of TILs between Ki67-high and Ki67-low breast cancers remains to be elucidated in a large series of patients. Similarly, negative hormone receptors were associated with higher counts of CD8+ TILs, reflecting the known distinct immunogenic properties of triple-negative and HER2-enriched breast cancers. These findings are consistent with the majority of the literature regarding correlations between TILs and clinicopathological parameters [38,39,40].

Regarding efficacy, this study confirmed the favorable role of high CD8 TILs in BC prognosis. The prognostic role of TILs in BC has been widely reported [41,42,43], with a proven impact on survival [44] and rates of complete response [45]. However, it is not yet clear which subsets of TILs contribute more to this outcome. TILs in BC are largely composed of CD8+ and, to a lesser extent, CD4+ T cells, macrophages, mast cells, regulatory T cells, and plasma cells [46]. CD8+ cytotoxic T cells are known to play a crucial role in the host’s adaptive immune response against cancer and kill cancer cells through several mechanisms [41,47]. In our study, patients with high counts of total CD8+ TILs had longer DFS and OS compared to those with low counts/mm^2^, reflecting their widely reported positive impact on patient outcomes [15,16].

In recent years, research efforts have focused on establishing the specific population of TILs that could more precisely, and with repeatability, predict clinical outcomes, as the cell type and location of TILs seem to be of great interest. Two TIL locations, stromal and intratumoral, are being investigated separately. This research field is where most discrepancies and ambiguous results appear. In the present study, patients with high counts/mm^2^ of iCD8 demonstrated DFS and OS benefits. This finding is in line with several published studies, including one by our research group, which studied BC patients in adjuvant settings [40,48,49] and contradicts the results of Catacchio et al. [38]. On the other hand, only a trend towards improved DFS was observed for tumors with high counts/mm^2^ of sCD8, despite the fact that in most published studies, this lymphocytic subset has been associated with a statistically significant survival benefit. These contradictory results can be explained by a variety of reasons, such as cancer heterogeneity, treatment setting, different methodology, and variability in patients’ populations between studies, along with interobserver discrepancies. In contrast to another published study of our group by Koletsa et al. [40], in the present study, sTIL density did not affect either DFS or OS.

In addition to the above-mentioned factors, the wide distribution of TILs counts/mm^2^, which led us and other researchers to use binary instead of continuous variables; the cell heterogeneity within a tumor specimen; the selection of tumor areas for evaluation; and the fact that TMAs represent a static snapshot of TILs, while the immune response in tumor stroma is a dynamic situation, are the main limitations of this study that should be considered.

An interesting observation of our study was that high CD8+ TILs are not prognostic in the first two to four years, and their favorable impact on DFS and OS appears later, as demonstrated in the Kaplan–Meier curves in Figure 2. At present, the relationship between intratumoral T cells numbers and patient prognosis is not simply a linear correlation, but rather a complicate phenomenon, in which T cells should be considered as a modifier of tumor growth. It has been shown in mice that T cells and tumor-reactive T cells are prone to gradient differentiation towards a dysfunctional state, and the degree of dysfunctionality may play a crucial role in immunosurveillance [50]. Moreover, Savas et al. [51], using single-cell RNA sequencing, demonstrated that the gene expression significance of CD8+ T_RM_ (T cells with features of tissue -resident memory) was associated with improved survival of patients with breast cancer and specifically in those with a triple-negative phenotype. According to these factors, an explanation of the aforementioned findings could be the gradient degree of immunosurveillance of T cells on prognosis over time. Nevertheless, it could be due to other unidentified reasons or chance, given the relatively small number of patients. Another limitation of this study, which obstructed our attempt to assess the impact of the TIL subset on patient survival according to BC molecular subtypes, was the small number of events of interest in the category of patients with high counts/mm^2^ of sCD8, iCD8, and total CD8. Only the luminal B subgroup could be analyzed, but without statistically significant results. However, given the strong impact on DFS and OS that we found in the entire patient cohort, we could indirectly suggest that HER2-enriched and triple-negative subgroups were, to a large extent, present in these results. This conjecture is consistent with the known prognostic role of TILs in these subgroups [6,9,13,17].

Although the molecular subtypes did not differ in survival in our study, specific clinicopathological characteristics were associated with improved outcomes in favor of patients with high CD8+ TIL counts and regardless of subsets. These were predominantly tumor size, nodal status, and histological grade, likely following the reported knowledge that the more advanced and aggressive tumors offer a biological background for neoantigen production. This, in turn, enhances a tumor’s immunogenicity, and intratumoral/stromal lymphocytic infiltration is able to predict better responses [52,53]. The role of tumor size and nodal size in prognosis is a well-known issue, and its importance to the high expression of TILs has previously been emphasized in other reports [13,54]. Overall, as more potentially prognostic/predictive factors appear, we need to be cautious about prognosis assessment, as it is not clear if the presence of high TILs could overcome the inferior prognosis that nodal status or tumor size intimate. Even from a biological point of view, high TILs are not always correlated with better outcomes. For instance, it has been reported that CD4+ TILs may change from effectors to suppressors when cancer progresses. This process may coincide with a substantial reduction in antigen expression, resulting in tumor tolerance and, therefore, progression. This negative regulatory role of CD4+ TILs needs to be distinguished from the conventional role of activated CD4+ TILs [44].

As intensified CMF chemotherapy is obsolete in early breast cancer, the regimen used represents another limitation of this study, along with its non-randomized design. On the other hand, we consider the long-term follow-up and the fact that all samples were prospectively collected and centrally assessed as strong points of our study.

In regards to safety, 93.6% of patients completed study treatment with multiple reasons for discontinuation, and no treatment-related deaths were reported. Despite the prophylactic use of G-CSF after every chemotherapy cycle, neutropenia was the most common adverse event, followed by peripheral neuropathy and fatigue. These toxicity data were similar to those previously reported from other randomized adjuvant breast cancer clinical trials conducted by our group [25,26,55].

## 4. Materials and Methods

### 4.1. Patients

Eligible patients were at least 18 years old with node-positive early BC, or node-negative disease of intermediate risk according to the 2005 St. Gallen criteria [56]. The patients had undergone breast-conserving surgery or modified radical mastectomy with tumor-free margins, and had adequate hematologic, hepatic, and renal function, a performance status of 0 to 1 on the Eastern Cooperative Oncology Group (ECOG) scale, and presented no evidence of serious cardiac disease (normal left ventricular ejection fraction [LVEF] demonstrated by a multiple gated acquisition (MUGA) scan or echocardiogram was a mandatory procedure).

Before enrollment, each patient signed a written informed consent form for the use of their biological material for future research purposes.

All investigations conducted in this study complied with the principles expressed in the Declaration of Helsinki.

The study protocol was approved by the Bioethics Committee of the Aristotle University of Thessaloniki, School of Medicine (77/10.6.14), by the Institutional Review Board of Papageorgiou Hospital (180/15.7.13), and by the Institutional Review Board of Thermi Clinic (307/2.3.16). The study was registered in the Australian New Zealand Clinical Trials Registry (ANZCTR) and allocated the following Registration Number: ACTRN12615000161527.

Pre-treatment evaluation included medical history, physical examination, imaging examinations according to international guidelines, complete blood count (CBC), and comprehensive biochemistry. Blood tests were obligatory before each chemotherapy cycle and EF, after the completion of chemotherapy, and then every four months during treatment with trastuzumab. Additionally, blood examinations were performed whenever clinically indicated (e.g., in cases of fever over 38 °C, severe stomatitis or diarrhea).

### 4.2. Treatment

The chemotherapeutic regimen of the present study consisted of three cycles of epirubicin (E, 110 mg/m^2^) every 2 weeks, followed by 3 cycles of paclitaxel (T, 200 mg/m^2^) every 2 weeks, followed by 3 cycles of intensified CMF (cyclophosphamide 840 mg/m^2^, methotrexate 57 mg/m^2^ and fluorouracil 840 mg/m^2^) every 2 weeks (E-T-CMF) with G-CSF support. According to the treatment protocol, no patient received preoperative treatment.

Dose modifications were performed as previously described [21]. Toxicity was assessed using the National Cancer Institute Common Terminology Criteria for Adverse Events, Version 3.0. Patients with HER2-positive tumors received 52 weeks of trastuzumab intravenously, initially at a dose of 8 mg/kg as a loading dose, and subsequently 6 mg/kg every three weeks, following the delivery of the last cycle of chemotherapy.

Radiation therapy (RT) was planned for all patients who had undergone partial mastectomy or those with tumor size ≥5 cm and/or more than 4 positive lymph nodes, regardless the type of surgery (conservative or radical), 3–4 weeks following the completion of chemotherapy.

Hormonal therapy was administered to all patients with hormone receptor-positive tumors. Details regarding the administration of hormonotherapy were previously reported [57].

### 4.3. Immunohistochemistry (IHC)

The immunohistochemical staining of all markers was performed on 3 μm ΤΜA sections using a Bond Max autostainer (Leica Microsystems, Wetzlar, Germany), as previously described [58] and shown in Supplemental Table S1.

IHC for estrogen receptor (ER), progesterone receptor (PgR), HER2, Ki67 labelling index, cytokeratin 5 (CK5), and epidermal growth factor receptor (EGFR) was performed centrally at the Laboratory of Molecular Oncology of the Hellenic Foundation of Cancer Research, Aristotle University of Thessaloniki (Thessaloniki, Greece), as previously described [58]. The results were interpreted by experienced breast cancer pathologists and blinded to the patient’s demographic and clinical data. Tumors were also assessed for these basic pathological characteristics at the local laboratory of the center where each patient was enrolled.

Tumors were classified according to well-known immunohistochemical markers using the IHC4 model [58,59]. They were also categorized into five distinguished molecular subtypes, as previously reported [60,61].

Immunohistochemistry (IHC) and fluorescence in situ hybridization (FISH) protocols for ER, PgR, HER2, and Ki67 assessments have previously been described [58]. Briefly, HER2 was scored on a four-point scale from 0–3, with intense membrane staining in >30% invasive tumor cells classified as positive (3+ staining) [62]. Cut-offs for ER and PgR were set at 1% positive nuclei [63] and 14% for Ki67 [64]. The simultaneous staining of ER and PgR was considered as one parameter (hormone receptor status, HRS). Ki67 was scored as a continuous variable (% of positively stained nuclei); the highest score for each TMA core was recorded. FISH evaluation was performed in 20 tumor nuclei [65]. The HER2 gene was categorized as amplified for HER2/CEP17 ratios ≥2.2 [62], or for mean HER2 copy numbers (MB) >6 [66]. The Topoisomerase 2a/Centromere 17 (TOP2A/CEN17) ratio cut-off for TOP2A amplification was ≥2.0 [67]. Lymphocytes expressing CD8 were detected using the monoclonal mouse antihuman antibody CD8 (clone C8/144B, code M7103. dilution 1:80) according to the protocol of the manufacturer (Dako, Glostrup, Denmark).

### 4.4. Evaluation of CD8+ Lymphocytes

CD8+ lymphocytes were evaluated by two Pathologists without knowledge of the clinical, pathological, and survival data.

Immunostaining for CD8 was estimated in the tumor stroma (sCD8), as well as in the intratumoral/intraepithelial compartment where CD8+ cells were attached to malignant cells (iCD8). In addition, CD8+ cells within the tumor area (total CD8 (tCD8)) defined as the sum of sCD8 and iCD8 was also examined.

For each core (1.5 mm of diameter), CD8+ cells were counted in four fields of magnification (×200) covering the entire area of the core. Therefore, four values per core were recorded. In cases in which the distinction of sCD8 and iCD8 was ambiguous, CD8 positivity was estimated at higher magnification (×400). The density of CD8+ cells in each tumor compartment was assessed as the ratio of cell counts per mm^2^ surface. Therefore, the stromal tumor area, the tumor area occupied by the malignant cells and the total tumor area were recorded as % increments of the total core area on matched Hematoxylin and Eosin TMA sections. The surface of each compartment in mm^2^ was calculated based on the percentage of the recorded area % and the total core surface (1.76625 mm^2^ for 1.5 mm cores). Then, the stromal CD8+ cell count per core was divided by the respective stromal surface, the intratumoral CD8+ cell count by the respective malignant cell surface, and the total tumor cell count by the total tumor surface, to assess the density of CD8 positive cells [36,68]. Average values were recorded in cases of tumors which were evaluated on multiple cores. The obtained values were distributed in an extremely wide range, while multiple outliers were identified for each lymphocytic subset, accounting for a significant percentage of the total sample in each case. These outliers were natural (i.e., they could not be attributed to technical causes). The inclusion of outliers in the continuous lymphocytic subset variables contributed to skewed analyses that were statistically inaccurate. Omitting them from the analysis, however, would have led to misinterpretation of the results. Therefore, we considered the upper quartile (75th percentile) of each distribution to be an appropriate threshold for the classification of tumors into high and low counts/mm^2^.

### 4.5. Evaluation of TILs

Whole sections of hematoxylin and eosin stained slides were used to evaluate stromal TILs (sTILs) in accordance to the criteria proposed by the International TILs BC Working Group. The percentage of all mononuclear cells (including lymphocytes and plasma cells) in the stromal tumor component within the border of invasive carcinomas was recorded. Stromal TILs density was assessed as an average of all evaluated ×100 fields per tumor.

### 4.6. Follow-Up

All patients were followed up at study entry, every six months for the first five years and every year thereafter, with clinical examinations, CBC, biochemistry panels, serological markers, chest X-rays, and abdominal ultrasonography (or CT scans if clinically indicated). Mammography and ultrasonography of the patients’ breasts were performed annually. Bone scans were not routinely performed after the third year, except when clinically indicated.

### 4.7. Statistical Analysis

Disease-free survival (DFS), calculated from the date of study treatment initiation to the first locoregional/distant relapse, contralateral breast cancer, secondary neoplasm, death from any cause, or last contact, whichever occurred, was the primary endpoint of the study. Secondary endpoints included assessment of overall survival (OS), estimated as the time interval from treatment initiation until death (from any cause) or last contact; the safety profile of the study treatment; and the prognostic significance of the biomarkers of interest for the patient’s outcome.

Due to the wide range of the distribution of stromal CD8 (sCD8), intratumoral (iCD8), and total CD8 (tCD8) and the presence of multiple natural outliers that could not be excluded from the analysis (to avoid misinterpretation of the results), the third quartiles of the respective distributions were used as the cut-off points to dichotomize tumors into high and low counts/mm^2^. Descriptive statistics with counts (%) and medians (minimum, maximum) values were used to summarize patient and tumor characteristics and the biomarker distributions. Associations between sCD8, iCD8, and tCD8 with selected clinicopathological parameters were assessed using a chi-square test (for categorical variables) and the Wilcoxon rank-sum test (for continuous variables). Spearman correlations were used to evaluate the associations of TILs with Ki67, sCD8, iCD8, and tCD8.

OS and DFS survival rates were obtained via Kaplan–Meier analyses and compared between groups with a two-sided log-rank test. Univariate Cox regression models were applied to evaluate the effect of clinicopathological parameters of interest, CD8 and TILs on DFS and OS. In the multivariate analysis, the effect of each lymphocytic subset marker that was univariately associated with patient outcomes was adjusted for menopausal status (premenopausal, postmenopausal, tumor size (≤2 cm, 2.1–5 cm, >5 cm), nodal status (0–3, ≥4), histological grade (I–II, III), and adjuvant radiotherapy (yes, no). Age and the type of surgery were not included in the multivariate models, due to their correlation with menopausal status and nodal status, respectively. All statistical analyses were performed using the SAS software (SAS version 9.4, SAS Institute Inc. Cary, NC, USA). Statistical significance was set at a two-sided *p* of 0.050.

## 5. Conclusions

In conclusion, the present trial showed that dose-dense E-T-CMF is a tolerable chemotherapy regimen for adjuvant treatment of intermediate/high risk breast cancer, with an efficacy comparable to more recent dose-dense schemes. CD8+ TILs, and especially their intratumoral subset, represent a potential favorable prognostic factor and impact survival rates. Although quantifying TILs using histopathology and immunohistochemistry is the most used technique, a variety of methodological factors may confound the results and, therefore, the impact of TILs in prognosis. In addition, the quantification of TILs does not take into account the dynamics and functionality of the tumor microenvironment. Studies in various histological subtypes of breast cancer, along with strict laboratory procedures and the use of novel approaches, such as automated computational assessments, are needed, in order to better understand the role of TILs and their subsets in BC prognosis and treatment.

## Figures and Tables

**Table 1:** Patient and tumor characteristics.

Parameter	
**Age at Diagnosis (N = 979)**	
Median (min, max)	54.2(28.1, 82.7)
	**N (%)**
**Menopausal status at diagnosis (N = 979)**	
Premenopausal	415(42.4)
Postmenopausal	564(57.6)
**Surgery (N = 979)**	
Modified radical	545(55.7)
Partial mastectomy	434(44.3)
**Tumor size (N = 978)**	
≤2 cm	395(40.4)
2.1–5 cm	506(51.7)
>5 cm	77(7.9)
**N of positive nodes (N = 975)**	
0	303(31.1)
1–3	378(38.8)
≥4	294(30.2)
**Grade (N = 978)**	
1	70(7.2)
2	456(46.6)
3	452(46.2)
**Histological classification (N = 978)**	
Invasive ductal carcinoma	829(84.8)
Invasive lobular carcinoma	93(9.5)
Carcinoma with medullary features	12(1.2)
Mixed	19(1.9)
Mucinous carcinoma	4(0.41)
Invasive solid papillary carcinoma	4(0.41)
Tubular carcinoma	3(0.31)
Other (specify)	14(1.4)
ER/PgR (N = 978) *	
Negative	230(23.5)
Positive	748(76.5)
HER2 status (N = 976) *	
Negative	751(76.9)
Positive	225(23.1)
**Adjuvant HT (N = 972)**	
No	251(25.8)
Yes	721(74.2)
**Adjuvant RT (N = 967)**	
No	259(26.8)
Yes	708(73.2)
**Trastuzumab administration (N = 978)**	
No	763(78.0)
Yes	215(22.0)

* Based on local assessment. N, number.

**Table 2:** Results of Cox multivariate regression models for DFS and OS in the entire cohort.

Parameter	Median (Range)	Event/Total	HR (95% CI)	*p*-Value	Event/Total	HR (95% CI)	*p*-Value
		DFS			OS		
iCD8							
High	26.63 (12.02–661.21)	27/155	0.59 (0.39–0.91)	0.016 *	23/155	0.61 (0.39–0.98)	0.039 **
Low	1.97 (0.00–12.07)	128/461	Reference	--	101/461	Reference	--
tCD8							
High	150.12 (94.94–726.46)	30/155	0.66 (0.44–0.98)	**0.041 ^^^**	22/155	0.56 (0.35–0.90)	**0.015 ^^^^**
Low	35.48 (0.57–94.13)	125/461	Reference	--	102/461	Reference	--
sCD8							
High	339.09 (222.79–1961.38)	29/152	0.64 (0.43–0.97)	**0.036 #**	26/152	0.64 (0.40–1.00)	**0.05 ##**
Low	78.45 (1.89–221.32)	126/464	Reference	--	103/464	Reference	

The following clinicopathological parameters were statistically significant in the examined multivariate models: * menopausal status (*p* = 0.044), tumor size (*p* = 0.017), nodal status (*p* < 0.001), ** menopausal status (*p* = 0.030), tumor size (*p* = 0.049), nodal status (*p* < 0.001), histological grade (*p* = 0.008), ^ menopausal status (*p* = 0.033), tumor size (*p* = 0.013), nodal status (*p* < 0.001), ^^ menopausal status (*p* = 0.024), tumor size (*p* = 0.035), nodal status (*p* < 0.001), histological grade (*p* = 0.010), # menopausal status (*p* = 0.030), tumor size (*p* = 0.015), nodal status (*p* < 0.001), ## menopausal status (*p* = 0.023), nodal status (*p* < 0.001), tumor size (*p* = 0.035), histological grade (*p* = 0.010). (Bold are the *p*-values).

**Table 3:** Incidence of grade 3 and 4 adverse events recorded throughout the study treatment among the 944 patients with available data.

Adverse Event	Grade 3			Grade 4		
	N of Evts	N of Pts	%	N of Evts	N of Pts	%
Total	293	214	22.67	32	28	2.97
ALP	2	2	0.21	0	0	0
ALT	11	11	1.17	2	2	0.21
AST	2	2	0.21	0	0	0
Allergic reaction/hypersensitivity (including drug fever)	8	8	0.85	1	1	0.11
Amylase	1	1	0.11	0	0	0
Anorexia	1	1	0.11	0	0	0
Bilirubin (hyperbilirubinemia)	1	1	0.11	0	0	0
Calcium, serum low (hypocalcemia)	3	3	0.32	1	1	0.11
Constipation	1	1	0.11	0	0	0
Diarrhea	2	2	0.21	0	0	0
Fatigue (asthenia, lethargy, malaise)	24	24	2.54	0	0	0
Febrile neutropenia (fever of unknown origin without clinically or microbiologically documented infection) (ANC <1.0 × 10^9^/L, fever > = 38.5 °C)	12	12	1.27	2	2	0.21
GGT	21	21	2.22	0	0	0
Glucose, serum high (hyperglycemia)	5	5	0.53	0	0	0
Hemoglobin	5	5	0.53	0	0	0
Hepatobiliary/Pancreas- Other (Hepatotoxicity)	1	1	0.11	0	0	0
Infection with unknown ANC: Upper airway NOS	2	2	0.21	0	0	0
Infection site reaction/extravasation changes	1	1	0.11	0	0	0
Infection with unknown ANC: Blood	0	0	0	1	1	0.11
Infection- Other	2	2	0.21	0	0	0
LDH	3	3	0.32	0	0	0
Leucocytes (total WBC)	15	15	1.59	3	3	0.32
Lymphopenia	2	2	0.21	1	1	0.11
Magnesium, serum-high (hypermagnesemia)	1	1	0.11	0	0	0
Mucositis/stomatitis(functional/symptomatic)	4	4	0.42	1	1	0.11
Nausea	6	6	0.64	0	0	0
Neurology-Other (Instability)	1	1	0.11	0	0	0
Neuropathy: motor/sensory	45	45	4.77	0	0	0
Neutrophils/granulocytes (ANC/AGC)	51	51	5.4	14	14	1.48
Pain: Abdomen NOS	2	2	0.21	0	0	0
Pain: Bone	13	13	1.38	0	0	0
Pain: Joint	1	1	0.11	0	0	0
Pain: muscle	12	12	1.27	0	0	0
Phlebitis (including superficial thrombosis)	1	1	0.11	0	0	0
Phosphate, serum-low (hypophosphatemia)	2	2	0.21	0	0	0
Platelets	0	0	0	1	1	0.11
Potassium, serum-high (hyperkalemia)	0	0	0	1	1	0.11
Potassium, serum-low (hypokalemia)	3	3	0.32	0	0	0
Pruritus/itching	5	5	0.53	1	1	0.11
Rash: hand-foot skin reaction	6	6	0.64	0	0	0
Syncope (fainting)	1	1	0.11	0	0	0
Thrombus/embolism	2	2	0.21	0	0	0
Uric acid, serum-high (hyperuricemia)	1	1	0.11	3	3	0.32
Vascular other-thrombosis arterial leg	1	1	0.11	0	0	0
Vomiting	6	6	0.64	0	0	0

## Data Availability

The data presented in this study are available in the article and supplementary material, while further details can be obtained on request from the corresponding author.

## Supplementary Materials

The following supporting information can be downloaded at: https://files.hecog.gr/Spathas_manuscript_Supplemental_.docx. The supplemental data presented in this study (Supplemental Figure S1 Distribution of sCD8, iCD8 and total CD8.; Supplemental Table S1. Staining protocols and manufacturers for IHC markers. ER1: citric acid-pH6, ER2: EDTA-pH 9, enz2: Proteinase K, RT: Room Temperature; Supplemental Table S2. Association of iCD8, sCD8 and tCD8 with selected clinicopathological parameters of interest; Supplemental Table S3. Sites of relapse in the entire cohort of patients; Supplemental Table S4. Association of clinicopathological parameters of interest with patient outcome; Supplemental Table S5. Association of sCD8, iCD8, and total CD8 with DFS and OS in the entire cohort of patients and among patients with luminal B tumors) are available in https://files.hecog.gr/Spathas_manuscript_Supplemental_.docx.
